# Tumour lysis syndrome in the setting of a high-grade B-cell lymphoma with primary bone marrow localization – a case report

**DOI:** 10.11613/BM.2026.020901

**Published:** 2026-04-15

**Authors:** Iva Ivanko, Josipa Josipović, Ivana Ćelap, Sabina Novaković Coha, Hana Matijaca, Tomislav Brblić, Sandra Kojić Katović, Petar Gaćina

**Affiliations:** 1 Department of Haematology, University Hospital Centre Sestre milosrdnice, Zagreb, Croatia; 2 School of Medicine, Catholic University of Croatia, Zagreb, Croatia; 3 Department of Nephrology and Arterial Hypertension, University Hospital Centre Sestre milosrdnice, Zagreb, Croatia; 4 Department of Clinical Chemistry, University Hospital Centre Sestre milosrdnice, Zagreb, Croatia; 5 Faculty of Pharmacy and Biochemistry, University of Zagreb, Zagreb, Croatia; 6 Department of Pathology and Cytology, University Hospital Centre Sestre milosrdnice, Zagreb, Croatia; 7 School of Dental Medicine, University of Zagreb, Zagreb, Croatia

**Keywords:** tumour lysis syndrome, acute kidney failure, non-Hodgkin lymphoma

## Abstract

Tumour lysis syndrome (TLS) is an oncologic emergency usually arising after chemotherapy administration, caused by a massive tumour breakdown and characterized by a specific laboratory and clinical findings. We present a case of TLS, both spontaneous and treatment related, in a 59-year-old woman with high grade B-cell lymphoma without apparent bulky disease on radiologic scans. The patient presented with acute kidney failure with marked hyperuricemia (2764 µmol/L) and extremely high lactate dehydrogenase (LD) activity (13,770 U/L). She was treated with rasburicase, corticosteroid pre-phase therapy, sequential chemotherapy and renal replacement therapy tailored to electrolytic disturbances. After initial resolution of spontaneous TLS, at the start of chemotherapy administration, she experienced systemic inflammatory response (SIRS) and therapy related TLS which included, amongst else, vast hyperphosphatemia (6.12 mmol/L). Despite all intensive care treatment, patient went into multiorgan failure preceding lethal outcome. This case report confirms the value of laboratory parameters in establishing the diagnosis of TLS with LD as an indicator of massive tumour turnover. Due to a high risk of deleterious complications, management of extreme TLS cases challenges the current guidelines of TLS therapy. Modern treatment options such as targeted anticytokine therapy could potentially bring benefit in these specific circumstances.

## Graphical abstract

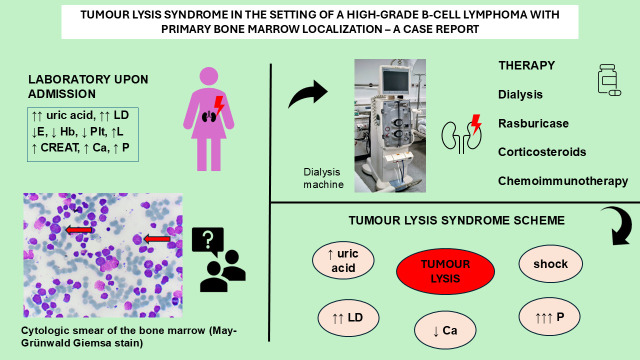

## Highlights

• Tumour lysis syndrome (TLS) is a grave concern in aggressive lymphoma treatment

• High-grade B-cell lymphoma is amongst leading cause of TLS

• Laboratory parameters can predict the magnitude of TLS

• Extreme cases of TLS may require management beyond standard treatment guidelines

## Introduction

Tumour lysis syndrome (TLS) is an oncologic emergency characterized by a specific laboratory abnormalities (hyperkalemia, hyperphosphatemia, hyperuricemia and hypocalcemia) often coupled with clinical signs of multiple organ dysfunction (kidney damage, cardiac arrythmia, sudden death, seizure) because of a fast tumor degradation (*1*). In most cases, TLS occurs during or immediately after delivery of a targeted tumor therapy, although spontaneous events have been described (*2*, *3*). Predominately, malignant hematological neoplasms such as high-grade lymphoproliferative neoplasms (Burkitt lymphoma) carry significant risk of TLS but other non-hematologic, solid tumors can pose a serious threat (*4*).

Here, we describe a case of TLS occurring in the setting of high-grade B-cell non-Hodgkin lymphoma characterized with a profound, life-threatening electrolytic disturbances and ominous clinical course.

## Case presentation

**Figure 1:**
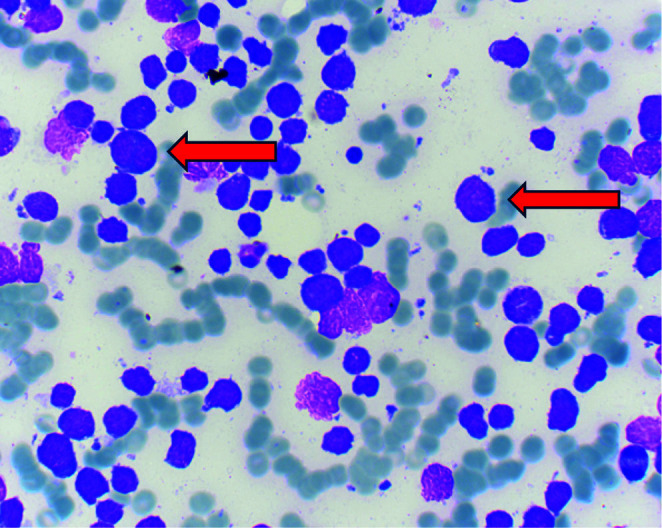
Cytologic smear of the bone marrow aspirate (May-Grünwald Giemsa stain) of a patient with high-grade B-cell lymphoma. Red arrows indicate an example of the high-grade B-cell lymphoma cell (malignant transformation of B-lymphocytes).

Fifty-nine-year-old female patient with a history of a well-controlled arterial hypertension and insulin non-dependent diabetes mellitus, was admitted to the Department of Hematology, Clinical Hospital Centre Sestre milosrdnice, Zagreb, Croatia, in April 2025, under the suspicion of acute leukemia. The patient was transferred to our unit from a local hospital. Initial laboratory findings revealed leukocytosis, anemia and thrombocytopenia requiring transfusion therapy, marked elevation of lactate dehydrogenase (LD) activity and signs of acute renal injury with hyperuricemia (Table 1). Peripheral blood smear (obtained in the local hospital) revealed 30% blasts. Upon admission to our hospital the patient presented with uncompromised vital signs (temperature, heart rate, respiration, blood pressure) with preserved diuresis. She reported worsening malaise for several days. During the first 24 hours of hospitalization, the patient’s renal function continued to decline with the development of anuria. After consulting the Department of Dialysis, the patient underwent continuous veno-venous hemodiafiltration (CVVHDF) in the Intensive Care Unit (ICU). Rasburicase treatment (0.20 mg *per* kg of total body weight, on day 2 and day 3, intravenously) commenced alongside dialysis in order to minimize further renal deterioration caused by extreme hyperuricemia. Cytological smear of the bone marrow, performed on the 2nd day of hospitalization, showed almost a 100% infiltration of atypical blasts without peroxidase and periodic acid-Schiff coloration arousing suspicion of an acute lymphoblastic leukemia (Figure 1). However, flow cytometry of the bone marrow aspirate indicated a diagnosis of a mature CD10+ B-lymphoproliferative disorder.

**Table 1:** Laboratory findings in a patient with high-grade B-cell lymphoma and tumour lysis syndrome

	**Day of hospitalization**					
**Laboratory parameter, unit** **(reference interval)**	**1st**	**2nd**	**6th**	**11th**	**12th** **(6 a.m.)**	**12th** **(6 p.m.)**
Creatinine, µmol/L (49-90)	488	541	145	282	255	189
Uric acid, µmol/L (134-337)	2764	2709	235	1379	668	/
Phosporus, mmol/L (0.79-1.42)	1.92	/	1.2	3.24	4.76	6.12
Potassium, mmol/L (3.9-5.1)	4.7	4.5	3.3	5.1	6.1	7.4
Calcium, mmol/L (2.14-2.53)	2.69	/	2.30	1.9	1.75	1.56
LD, U/L 37 °C (124-241)	> 7325	> 7325	> 7325	13,770	> 7325	> 7325
Leukocytes, x10^9^/L (3.4-9.7)	25.9	23.3	38.5	3.7	0.8	1.0
Hemoglobin, g/L (119-157)	80	80	88	68	85	94
Platelets, x10^9^/L (158-424)	22	44	14	11	11	54
CRP, mg/L (< 5)	42.4	38.3	12.5	86.4	174	/
1st day - parameters reflect spontaneous tumor lysis syndrome, 11th day - parameters reflect onset of therapy related tumor lysis syndrome. LD - lactate dehydrogenase (for LD values greater than 7325 U/L to be expressed, the blood specimen requires additional technical method of blood specimen dilution). CRP - C-reactive protein.						

CD10+ non-Hodgkin lymphomas, predominately follicular lymphoma, were known to cause a high tumor burden but due to the clinical and laboratory findings, a transformation to a higher-grade lymphoma was anticipated from the bone marrow biopsy. Due to technical reasons this part of diagnostic evaluation was still ongoing.

While waiting for a definitive diagnosis, corticosteroid pre-phase treatment was initiated with the aim of preparing the patient for a definitive chemotherapy treatment. To assess lymphoma staging, a computed tomography (CT) scan was performed, and no large tumor masses were found in the mediastinum nor abdomen, which translated to a conclusion that the entire tumor burden was within the bone marrow. There were no findings of renal pathology nor post-renal obstruction. Over the next few days, CVVHDF was continued and methylprednisolone (100 mg intravenously *per* day for 9 days). Hyperuricemia was resolved; however, the patient was still anuric. The patient was also treated with parenteral antibiotic therapy (piperacillin/tazobactam) due to positive blood culture for *Clostridium perfringens* and urine culture for *Klebsiella pneumoniae.* Approximately one week into hospitalization bone marrow biopsy evaluation was completed, resulting in a diagnosis of a high-grade B-cell lymphoma with almost 100 percent of bone marrow infiltration.

After pre-phase corticosteroid treatment and the resolution of laboratory signs of TLS, the next step was introduction of sequential chemotherapy with intermittent dialysis support due to anuria. On the 10th day of hospitalization, anti-CD20 monoclonal antibody (rituximab) was slowly delivered during 12-hours interval (375 mg/m^2^) followed by a single attenuated dose of vincristine (1 mg intravenously), without of the rest planned cytostatic therapy. Usually after pre-phase corticosteroid treatment, chemotherapy is applied in full dose and regimen, but in this particular case with extreme tumor burden, we decided to apply more conservative approach. While receiving immunotherapy, the patient experienced hypotension, chills and temperature rise. The immunotherapy was temporarily stopped and patient received corticosteroid and anti-inflammatory treatment (paracetamol) with fluids. After stabilization, rituximab was started at lower infusional rate. During chemoimmunotherapy therapy administration diuresis was established (more than 1000 mL of urine output). However, laboratory evaluation on the 11th day revealed renal function worsening with rising hyperuricemia, hyperphosphatemia and hypocalcemia. Leukocyte count dropped from 30x10^9/^L to 3.7x10^9^/L in less than 24 hours indicating a fast tumor breakdown (Table 1). To comprehend the magnitude of TLS, we kindly asked Department of Clinical Chemistry for a definitive LD measure. On the 11th day of hospitalization, after nine days of pre-phase corticosteroid therapy, LD was 13,770 IU/L. The patient was clinically stable, and a course of intermittent hemodialysis was performed to obtain a fast control over electrolytic disturbances. Rasburicase was also repeated on the 11th day in a standard dose.

Unfortunately, progressive electrolyte and metabolic disturbances within the context of TLS developed, including extreme hyperphosphatemia along with hyperkalemia and hypocalcemia, ultimately leading to full-blown clinical TLS manifested by dyspnea and hypotension.

The patient was intubated; mechanically ventilated and arterial blood pressure was maintained by vasopressors. Continuous renal replacement therapy was commenced once again in the hope that continuous clearance of degrading products from the tumor cells would ease electrolyte disturbances. Despite all intensive care methods, the patients succumbed to multi-organ failure on the 12th day of hospitalization.

Permission for this case report to be published was given by the patient’s children.

## Laboratory methods

Venous blood for routine analysis was drawn according to patient management protocol in vacutainers with gel (for biochemistry tests, volume 5 mL) and with EDTA anticoagulant (for full blood count, volume 3 mL) (Greiner Bio-One, Kremsmünster, Austria). Biochemistry parameters (creatinine, LD, C-reactive protein, uric acid, phosphorus, potassium and calcium) were measured on biochemistry analyzer Alinity c (Abbott Diagnostics, Abbott Park, USA) using original reagents and according to manufacturer instructions. Full blood count was determined on Sysmex XN1000 (Sysmex Co, Kobe, Japan). Cytological bone marrow and peripheral blood analysis was done with May-Grünwald Giemsa stain on Olympus BX 41 microscope (Olympus, Hamburg, Germany).

## Discussion

We have presented a patient with high-grade B lymphoma whose illness encompassed two clinical and laboratory entities – spontaneous and treatment related TLS. While spontaneous TLS was characterized by extreme hyperuricemia, hallmark of treatment related TLS were extreme electrolytic disturbances, predominately hyperphosphatemia.

Tumor lysis syndrome is a well-known oncologic emergency with a high mortality rate (*5*, *6*). The hallmark of TLS is the release of massive quantities of cellular breakdown products leading to hyperuricemia which may contribute to acute kidney injury through intratubular urate crystal precipitation and obstruction (*7*).

The diagnosis is based on Cairo-Bishop definition of TLS which includes laboratory criteria of electrolyte abnormalities (hyperkalemia, hyperphosphatemia, hypocalcemia) with uric-acid elevation and clinical criteria (kidney damage, cardiac arrythmia, sudden death, seizure) (*1*).

Current treatment guidelines place focus on the use of hyperuricemia lowering agents, maintaining electrolyte equilibrium and fluid balance (*8*, *9*).

Rasburicase (recombinant urate oxidase), in the context of TLS, is a superior method of hyperuricemia management than allopurinol due to the uric acid degradation into much more water-soluble compound allantoin (*8*). It is worth mentioning that the blood specimen for uric acid monitoring after the usage of rasburicase should be collected in a prechilled tube, placed on ice, and the assay completed within four hours (*10*). Continuous degradation of uric acid *ex-vivo* can result in a falsely low concentration and affect the clinical decision for the rasburicase reusage.

Unlike hyperuricemia, severe hyperphosphatemia can only be resolved by a renal replacement therapy, especially CVVHDF due to better tolerance and hyperphosphatemia rebound avoidance (*8*, *11*).

Lactate dehydrogenase activity is not included in diagnostic criteria of TLS, however it points to a rapid tumor turnover which is hallmark of aggressive hematological malignancies. Tumor lysis syndrome risk stratification does acknowledge LD elevation as a high risk for TLS occurrence (*8*).

Our patient presented with spontaneous TLS based on hyperuricemia and hyperphosphatemia as the laboratory criterion and kidney failure as the clinical criterion by the Cairo-Bishop definition. Treatment related TLS was diagnosed also based on a Cairo-Bishop definition with a new onset of hyperuricemia and hyperphosphatemia, emerging hypocalcemia and hyperkalemia and clinical deterioration, both directly related to chemoimmunotherapy administration (Table 1).

The patient was treated according to the current guidelines for TLS management (*8*, *12*). The rasburicase was chosen over allopurinol due to the extreme hyperuricemia. Volume overload, suggested by the guidelines, was not applied due to anuria. Instead, kidney replacing therapy, both intermittent and continuous, was administered as a method of correction of electrolyte disturbances and metabolic acidosis arising from renal injury while preserving fluid balance. Continuous renal replacement therapy was specifically directed to suppress immunochemotherapy related vast hyperphosphatemia which is in accordance with guidelines and treatment approach used in real clinical practice (*13*, *14*). Pre-phase corticosteroid treatment was initiated before introducing targeted immunochemotherapy to minimize the risk of treatment related TLS. Immunochemotherapy itself was administered sequentially for the same reason. Recent study of Mohamad *et al.* implies that even immunotherapy alone applied in fractionated manner (during several days) can lower the risk of therapy related TLS in highly aggressive lymphomas (*15*).

**Figure 2:**
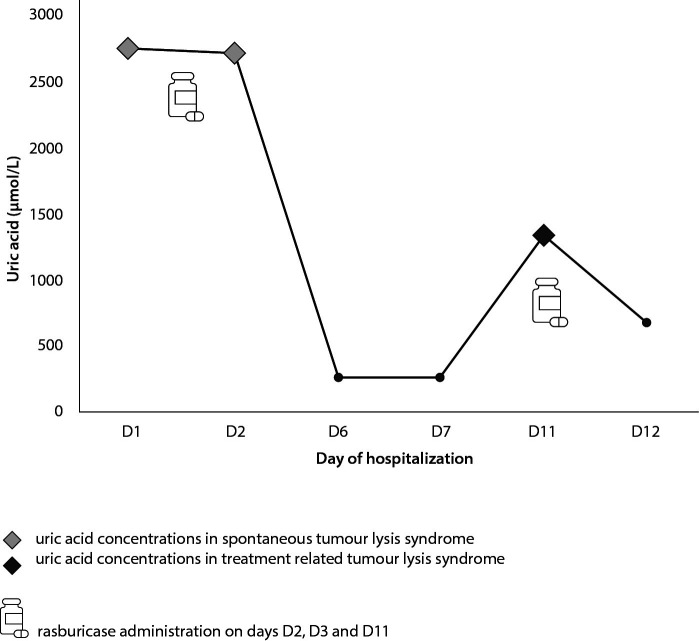
Dynamics of changes in acid uric concentration in patient with high-grade B-cell lymphoma by hospitalization day.

However, this case has proved to be an extreme presentation of TLS, where standard treatment methods, suggested by the guidelines, failed to suppress ominous circle of tumor degradation and organ injury. What is interesting is that only laboratory changes – hyperuricemia and severe hyperphosphatemia foreshadowed the magnitude of TLS with extreme LD elevation being an ominous sign of a rapid tumor turnover. Diagnostic radiology workup, commonly used as a measure of a tumor burden in lymphomas, had no value in this case due to the fact that there was no visible bulky disease. Dynamic of uric acid concentrations clearly indicate two phases of disease trajectory – spontaneous and treatment related TLS (Figure 2).

Further, patient’s reaction at the start of immunotherapy (hypotension, high fever) was in fact systemic inflammatory syndrome (SIRS) based on cytokine releasing syndrome (CRS). Although we did not measure IL-6 at the time, clinical presentation and symptoms amelioration to corticosteroid therapy support our theory. Interleukine-6 antagonists, such as tocilizumab are not a part of guidelines for TLS and are reserved for management of CRS caused by chimeric antigen receptor T-cell therapy (CAR-T) and bispecific antibody therapy (*16*). In the context of these novel therapies, IL-6 antagonist is used as anti-inflammatory agent directed at the systemic response to the massive tumor breakdown. It has been shown that high IL-6 concentrations in aggressive B non-Hodgkin lymphoma correlate with high LD, bone marrow involvement and poor outcome (*17*). Cytokine profile determination, including IL-6, could be of use for acknowledging patients in high risk of TLS and CRS directed therapy could potentially bring benefit in the management of extreme TLS.

In conclusion, although treatment of this patient was according to the current guidelines of TLS management, it failed to prevent lethal outcome. This case highlights the continuous need for expanding and upgrading standard treatment guidelines, starting from early recognition of the magnitude of TLS to introducing possible novel therapies which could potentially be beneficial in lowering high complication rates in extreme TLS cases.

## Data Availability

The data generated and analyzed in the presented study are not publicly available to preserve individuals’ privacy, but are available from the corresponding author on request which will not include patient’s personal informations.
